# Supplementary material for: AMPK Activation Is Indispensable for the Protective Effects of Caloric Restriction on Left Ventricular Function in Postinfarct Myocardium
Source: Biology (Basel). 2022 Mar 16;11(3):448. doi: 10.3390/biology11030448 (PMC8945456; doi:10.3390/biology11030448)
Supplement: Supplementary file 1 [file biology-11-00448-s001.zip › biology-1599211-supplementary.pdf]

biology

MDPI

Supplement to: Niemann et al.: AMPK activation is indispensable for the protective effects of caloric restriction on left ventricular function in postinfarct myocardium

Figures

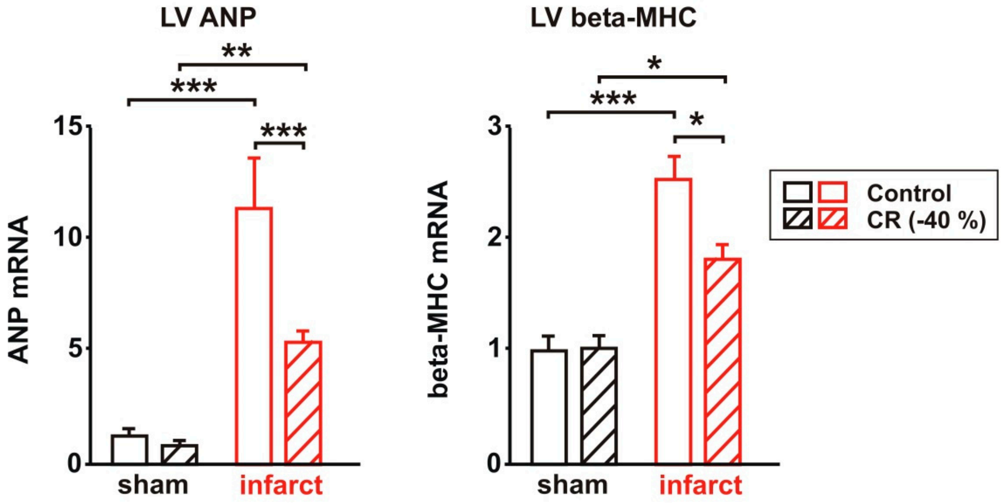

Figure S1. Effect of 8 weeks CR on the mRNA expression of molecular markers of hypertrophy. LV ANP and beta-MHC mRNA expression in sham or infarct rats following 8 weeks of control or CR diet. All data are mean±SEM. *n*= 6-7 animals per group, *: p<0.05; **: p<0.01; ***: p<0.001.

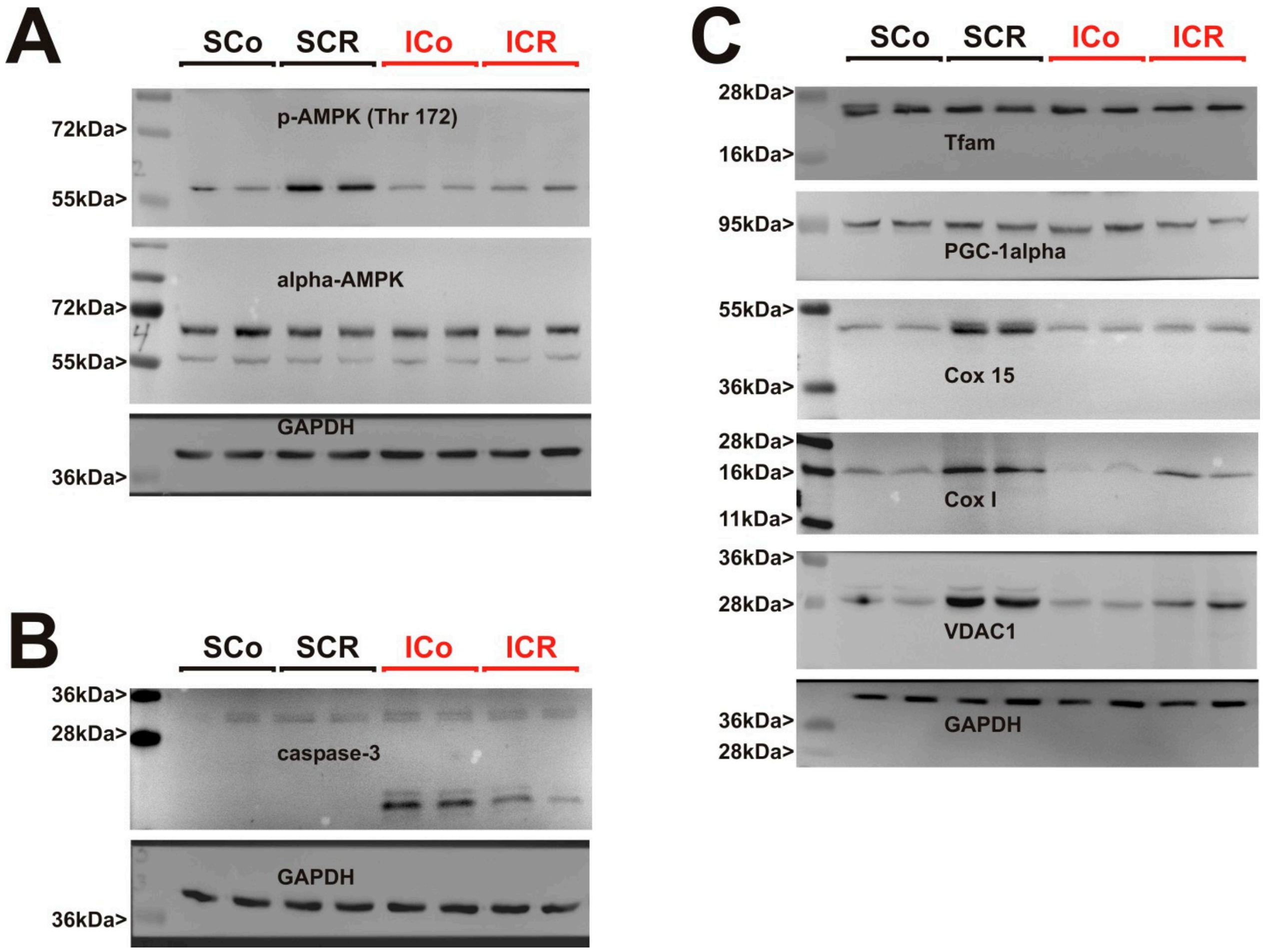

Figure S2. Effect of 8 weeks CR on AMPK activation, apoptosis and mitochondrial biogenesis. **A:** Full size representative Western blots of phosphorylation of AMPK (at Thr172) and alpha AMPK in the LV of sham or infarct rats following 8 weeks of control or CR diet. GAPDH served as loading control. **B:** Full size representative Western blots of full-length and large fragment (17/19 kDa) of cleaved, activated caspase-3 in the LV of sham or infarct rats following 8 weeks of control or CR diet. GAPDH served as loading control. **C:** Full size representative Western blots of Tfam, PGC-1alpha, mitochondrial components of complex IV (Cox 15 and Cox I) and VDAC1. GAPDH served as loading control.

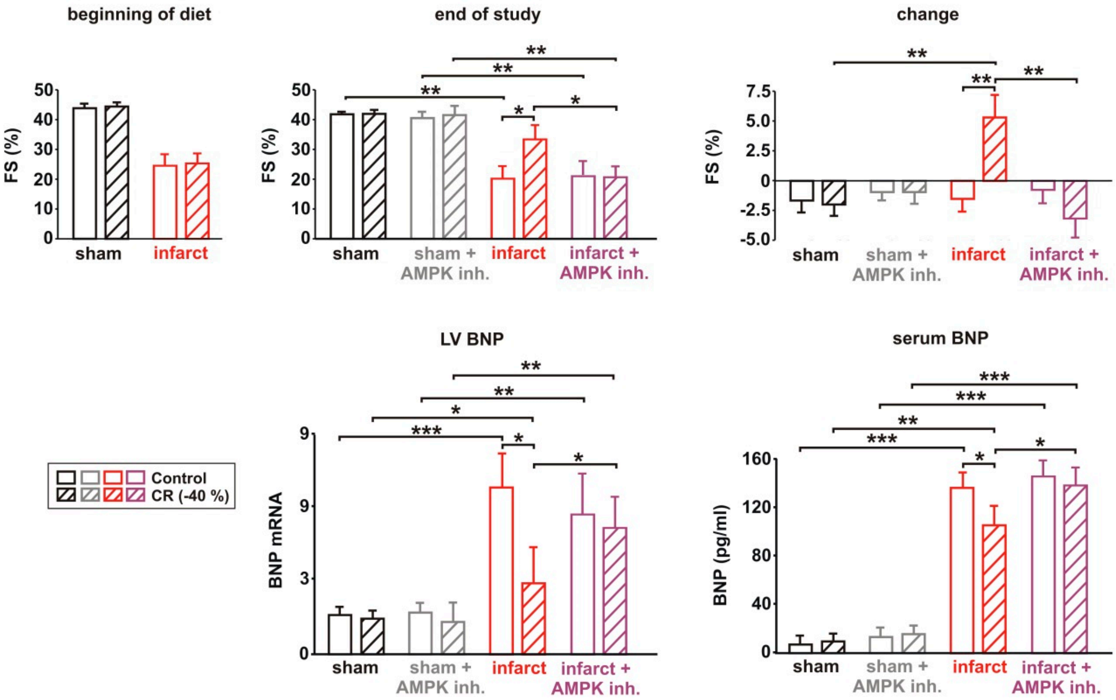

Figure S3. Effect of 8 weeks CR and 2 weeks of AMPK inhibition on LV systolic function. LV fractional shortening (FS%) at the beginning of the diet (upper left panel), at the end of the study (upper middle panel) and changes in FS during the feeding period (upper right panel) in sham or infarct rats following 8 weeks of control or CR diet. LV weight normalized to tibia length at the end of the study (lower left panel), LV mRNA expression (lower middle panel) and serum levels of brain natriuretic peptide (lower right panel) in sham or infarct rats following 8 weeks of control or CR diet. All data are mean±SEM. *n*= 6-7 animals per group, *: p<0.05; **: p<0.01; ***: p<0.001.

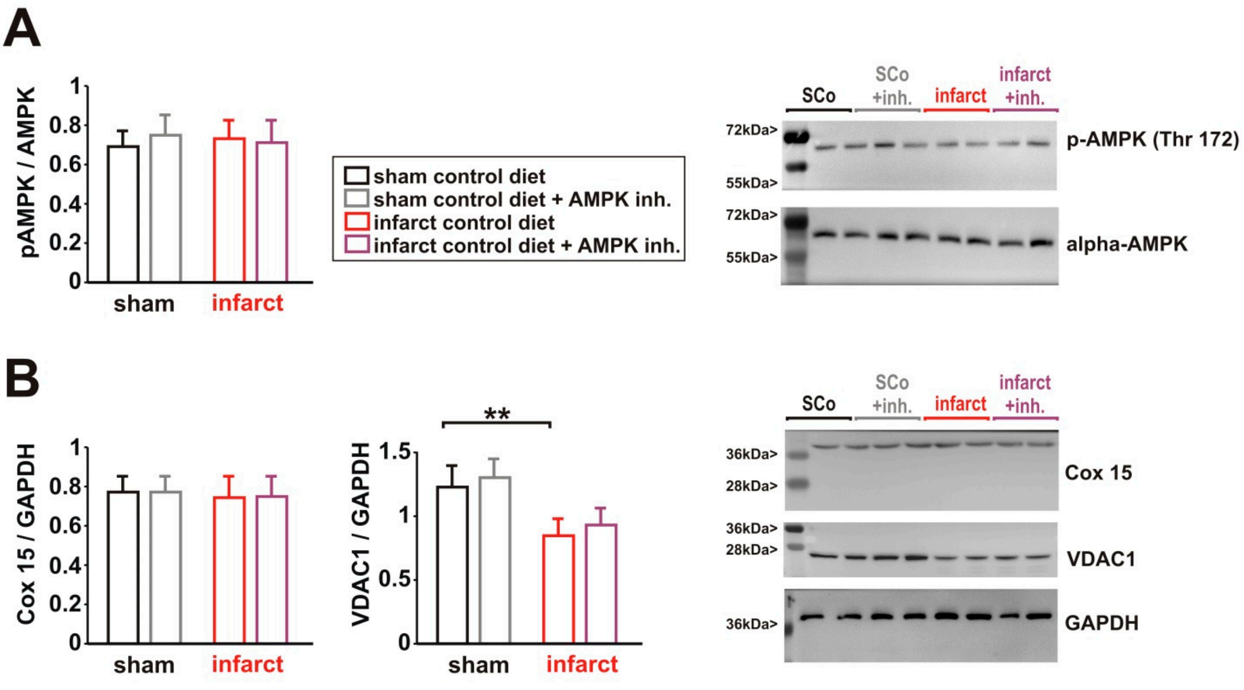

Figure S4. Effect of infarct and 2 weeks of AMPK inhibition on AMPK activation and mitochondrial biogenesis. **A:** Representative Western blots and quantification of phosphorylation of AMPK (at Thr172), normalized to alpha AMPK, in the LV of sham or infarct rats following 8 weeks of control diet and 2 weeks of AMPK inhibition. **B:** Representative Western blots and quantification of protein expression of Cox 15 and VDAC1, normalized to GAPDH, in LV tissue. All data are mean±SEM. n= 6-7 animals per group, *: p<0.05; **: p<0.01; ***: p<0.001.

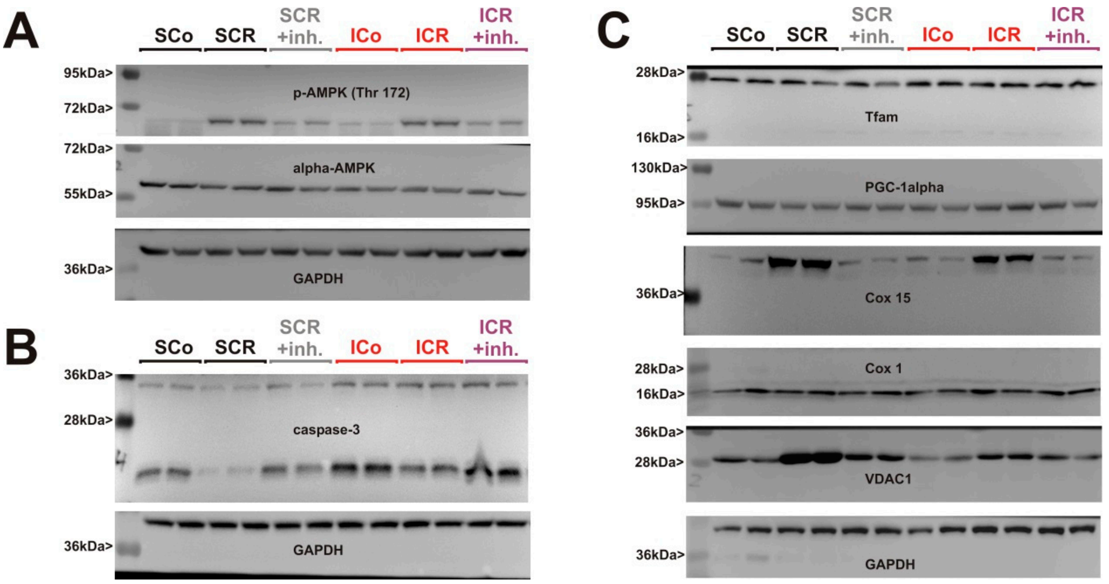

Figure S5. Effect of 8 weeks CR and 2 weeks of AMPK inhibition on AMPK activation, apoptosis and mitochondrial biogenesis. **A:** Full size representative Western blots of phosphorylation of AMPK (at Thr172) and alpha AMPK in the LV of sham or infarct rats following 8 weeks of control or CR diet and AMPK inhibition. GAPDH served as loading control. **B:** Full size representative Western blots of full-length and large fragment (17/19 kDa) of cleaved, activated caspase-3 in the LV of sham or infarct rats following 8 weeks of control or CR diet and AMPK inhibition. GAPDH served as loading control. **C:** Full size representative Western blots of protein expression of Tfam, PGC-1alpha, mitochondrial components of complex IV (Cox 15 and Cox I) and VDAC1 in LV tissue. GAPDH served as loading control.

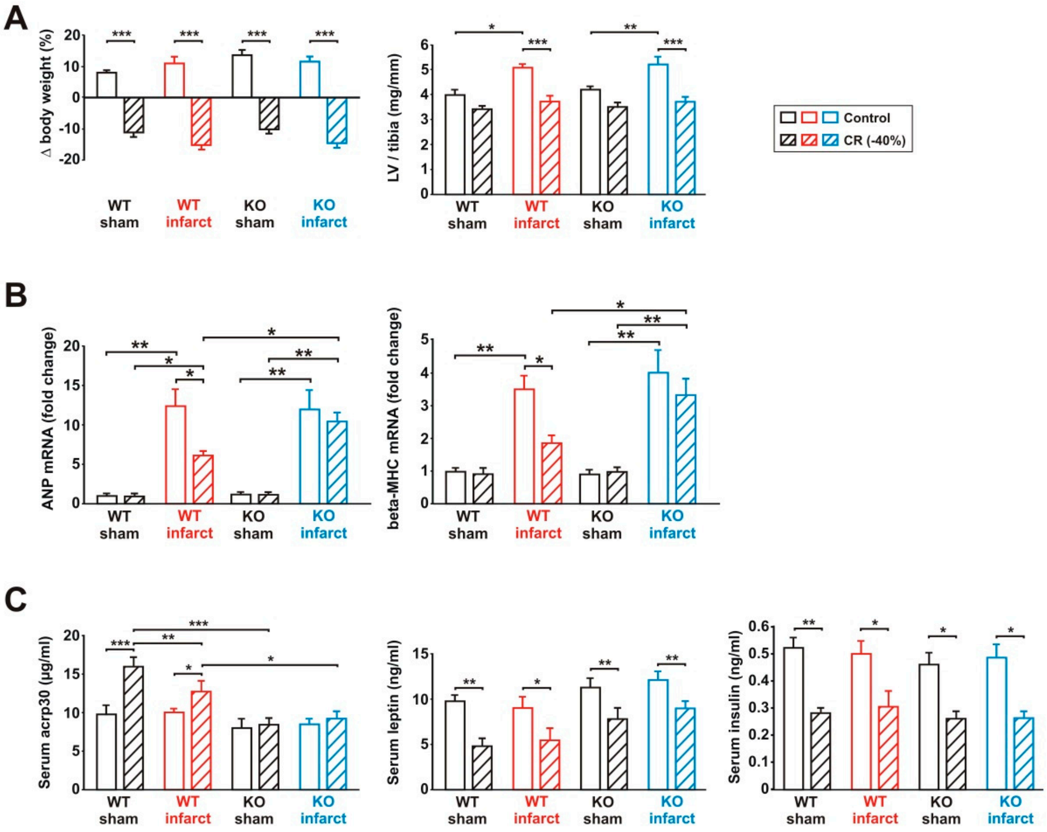

Figure S6. Effect of 8 weeks CR on body weight, LV weight, molecular markers of hypertrophy and plasma parameters. **A:** Change in body weight during the feeding period (left panel) and LV weight normalized to tibia length at the end of the study (right panel) in WT and KO sham or infarct mice following 8 weeks of control or CR diet. **B:** LV ANP and beta-MHC mRNA expression in WT and KO sham or infarct mice following 8 weeks of control or CR diet. **C:** Serum levels of acrp30, leptin and insulin in these mice, obtained after overnight fasting. All data are mean±SEM. *n*= 6-8 animals per group, *: p<0.05; **: p<0.01; ***: p<0.001.

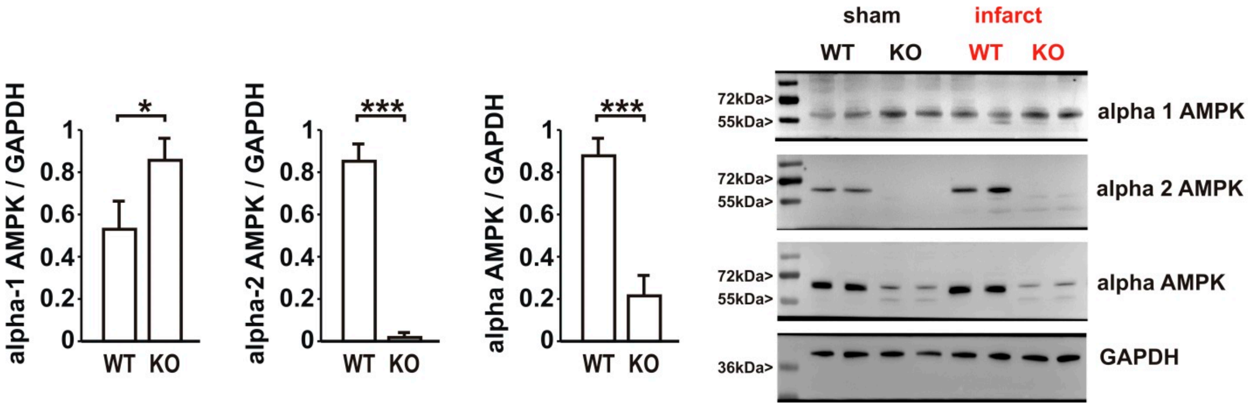

Figure S7. AMPK isoform expression in WT and KO mice. Representative full size Western blots and quantification of alpha 1 AMPK, alpha 2 AMPK and alpha AMPK in the LV of WT and KO sham or infarct mice. GAPDH served as loading control. All data are mean±SEM. *n*= 4 animals per group, *: p<0.05; ***: p<0.001.

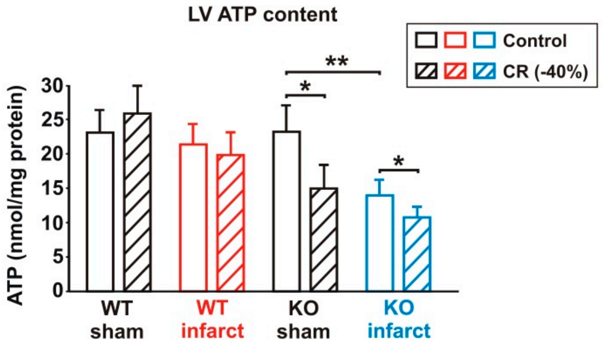

Figure S8. Quantitative analysis of myocardial ATP content. WT and KO sham or infarct mice were analyzed after 8 weeks of control or CR diet. All data are mean±SEM. n= 5 animals per group, *: p<0.05; **: p<0.01.

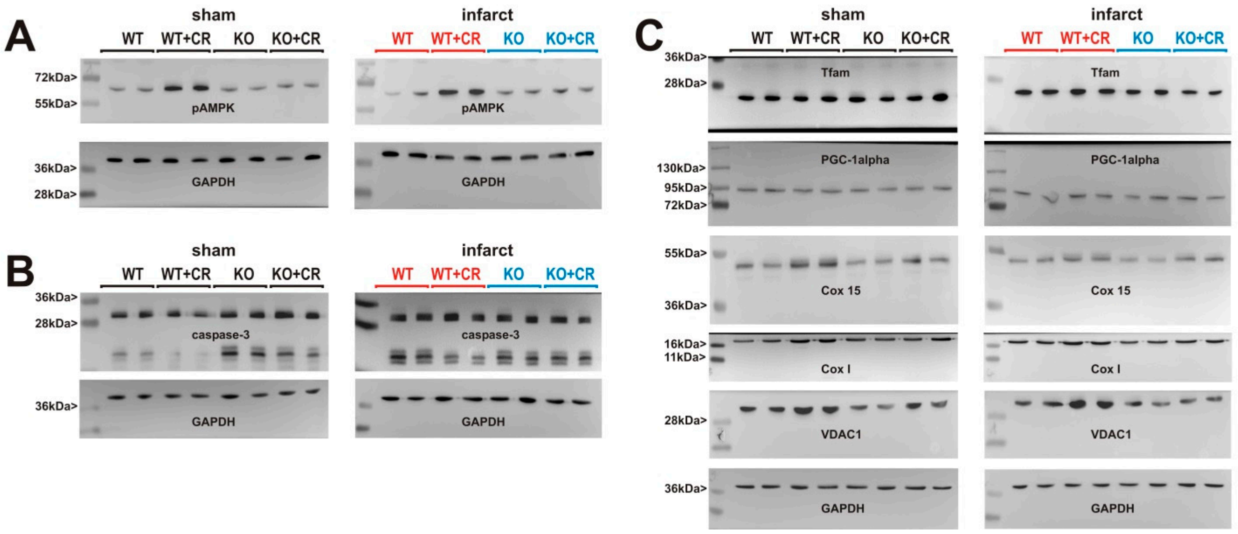

Figure S9. Effect of 8 weeks CR on AMPK activation, apoptosis and mitochondrial biogenesis. **A:** Full size representative Western blots of phosphorylation of AMPK (at Thr172) in WT and KO sham or infarct mice following 8 weeks of control or CR diet. GAPDH served as loading control. **B:** Full size representative Western blots of full-length and cleaved, activated caspase-3. GAPDH served as loading control. **C:** Full size representative Western blots of protein expression of Tfam, PGC-1alpha, mitochondrial components of complex IV (Cox 15 and Cox I) and VDAC1 in LV tissue. GAPDH served as loading control.
